# Effects of Repetitive Transcranial Magnetic Stimulation on Upper Limb Motor Function and Serum Lipid Metabolomics in Patients With Ischemic Stroke: A Randomized Controlled Study

**DOI:** 10.1002/brb3.70558

**Published:** 2025-05-26

**Authors:** Meng‐Meng Li, Fei‐Yang Jia, Peng‐Cheng Liu, Hong‐Ya Liu, Gui‐Juan Zhou, Xin‐Ke Peng, Jin‐Ling Wang, Shu‐Zhi Li, Jing Liu, Jun Zhou

**Affiliations:** ^1^ The First Affiliated Hospital, Rehabilitation Medicine Center, Hengyang Medical School University of South China Hengyang Hunan China; ^2^ The First Affiliated Hospital, Department of Rehabilitation, Hengyang Medical School University of South China Hengyang Hunan China; ^3^ The First Affiliated Hospital, Rehabilitation Laboratory, Hengyang Medical School University of South China Hengyang Hunan China; ^4^ The Affiliated Nanhua Hospital, Department of Rehabilitation, Hengyang Medical School University of South China Hengyang Hunan China

**Keywords:** ischemic stroke | lipidomics | repetitive transcranial magnetic stimulation | upper limb motor dysfunction

## Abstract

**Introduction:**

Repetitive transcranial magnetic stimulation (rTMS) can reduce upper extremity motor dysfunction in patients with stroke. However, the optimal parameters and mechanisms of rTMS in stroke treatment remain unclear. Therefore, this study aimed to investigate the protective effect and mechanism of rTMS at different frequencies on the motor function of the upper limbs in patients with cerebral infarction using lipid metabolomics methods.

**Methods:**

A total of 102 participants were randomly assigned to receive sham rTMS, 1 Hz rTMS, and 10 Hz rTMS. All participants were assessed at baseline and 2 weeks later using the Fugl‐Meyer Assessment upper extremity (FMA‐UE), National Institutes of Health Stroke Scale (NIHSS), Barthel scales, and serum collection—lipid metabolomics analysis of serum samples by untargeted metabolomics.

**Results:**

The improvement in FMA‐UE, NIHSS, and Barthel scores was more significant in 1 and 10 Hz rTMS groups than in the sham rTMS treatment group (*p* < 0.05). The improvement effect of FMA‐UE (*p* < 0.05) and Barthel (*p* < 0.05) was significantly more potent with 1 Hz rTMS than with 10 Hz rTMS. There was no significant difference in NHISS (*p* > 0.05). After rTMS treatment of patients with ischemic stroke, differential metabolites of serum lipids included diacylglycerol phosphoinositide, triacylglycerol, and dialkyl glycerol.

**Conclusion:**

Both low‐ and high‐frequency rTMS can effectively improve upper limb motor function and self‐care ability in patients with ischemic stroke. However, the effect of low‐frequency rTMS on improving upper limb motor function was more significant. Lipid metabolomics analysis revealed that high‐ and low‐frequency rTMS increased diacylglycerol phosphoinositide and triacylglycerol levels and reduced dialkyl glycerol levels.

## Graphical abstract

Both low‐ and high‐frequency rTMS enhance upper limb motor function/self‐care in ischemic stroke, with low‐frequency showing superior motor gains. Lipidomics revealed upregulated diacylglycerol phosphoinositide (DAG‐PI) and triacylglycerol (TAG) and reduced dialkyl glycerol (DAG) levels post‐intervention.

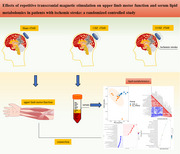

## 1 Introduction

Stroke is one of the top three causes of death and the leading cause of disability worldwide (Potter et al. 2022), with ischemic stroke accounting for approximately 87% of all stroke cases (Feigin et al. 2022). Stroke is a critical public health issue that imposes a significant burden on patients and their families, healthcare systems, and society (Ma et al. 2021). Ischemic stroke can cause severe brain damage due to an imbalance in blood supply, leading to various functional impairments (Wang et al. 2022). Among them, upper limb motor dysfunction (ULMD) exists in most stroke survivors, and the recovery rate is minimal (Nijland et al. 2010), significantly affecting the self‐care ability, quality of life (Kawakami et al. 2024), and career development of stroke patients (Błaż et al. 2022). However, no effective treatment is currently available. Interventions for ULMD may have a clinically meaningful impact on functional outcomes. Consequently, there is an urgent need for innovative and effective treatment strategies for ULMD after stroke.

Transcranial magnetic stimulation is a painless, noninvasive, and non‐intrusive treatment for brain stimulation (Klomjai et al. 2015). It generally achieves the goal of stimulating or inhibiting local cerebral cortical function by changing the stimulation frequency of transcranial magnetic stimulation (Bai et al. 2022). High‐frequency repetitive transcranial magnetic stimulation (rTMS) can summate excitatory postsynaptic potentials, causing abnormal neural excitability at the stimulation site (Tian and Izumi 2022). However, low‐frequency rTMS produces the opposite effect (Yuan et al. 2020). rTMS treats diseases by bidirectionally regulating the balance between excitatory and inhibitory brain functions (Notzon et al. 2018; Wada et al. 2022). The earliest and established clinical application of rTMS is the treatment of drug‐resistant depression by high‐frequency stimulation of the left dorsolateral prefrontal cortex, and it has been approved by the Food and Drug Administration (Gonsalves et al. 2022). Moreover, rTMS has broad development prospects in treating neurological diseases, including neuroinflammation (Attal et al. 2021) and stroke (Sheng et al. 2023). Clinical studies have revealed that rTMS can improve motor function and daily living ability in stroke patients (Veldema and Gharabaghi 2022).

Several smaller studies provide preliminary support for the feasibility and acceptability of rTMS as an intervention for individuals with ULMD in patients with ischemic stroke (Lv et al. 2023). rTMS can significantly improve the motor function of the upper limbs in patients with ischemic stroke (Du et al. 2019). The interhemispheric inhibition model demonstrated that low‐frequency rTMS on the unaffected side can improve upper limb function after stroke (Luk et al. 2022; Lv et al. 2023; Tosun et al. 2017). However, not all patients exhibit good benefits. The compensation model, which combines high‐frequency rTMS over the lesion, can also improve upper limb motor function after stroke (Chen, Lin et al. 2022; Chen, Yao et al. 2021). Furthermore, the mechanism by which rTMS improves motor function after stroke remains unclear.

The brain contains high concentrations of lipids, including cholesterol, sphingomyelin, and glycerophospholipids, due to the specific lipid flux regulated by the blood–brain barrier (Pifferi et al. 2021). These lipids are essential for energy storage, regulating membrane fluidity and permeability, and signal transduction (Castellanos et al. 2021). Previous studies have demonstrated that lipid metabolism can change significantly after ischemic stroke (Ma et al. 2022). After the onset of ischemia, polyunsaturated fatty acids are rapidly and abundantly released from the cell membranes of brain tissue, generating various short‐lived lipid mediators that protect brain tissue after a stroke (Kloska et al. 2020). In addition, an increase in the triglyceride‐to‐glucose index is associated with a higher risk of ischemic stroke in the general population (Yang et al. 2023). Lipid management is a crucial core strategy for the secondary prevention of ischemic stroke and is a vital management tool for patients with previous ischemic stroke (Kleindorfer et al. 2021). Consequently, considering the protective effect of rTMS on upper limb function in cerebral ischemia, we investigated whether serum lipid metabolism mediated the beneficial effects of rTMS on upper limb function in patients with ischemic stroke.

In this study, the efficacy of different rTMS frequencies on the recovery of upper limb function in patients with ischemic stroke was investigated. Simultaneously, we investigated whether there were differences in serum lipid metabolomics between ischemic stroke patients who received different frequencies of rTMS intervention and those who did not receive rTMS intervention using a non‐targeted lipidomics platform. Notably, this was the first study investigating the mechanism of lipid metabolomics in stroke patients treated with rTMS.

## 2 Methods

### 2.1 Design

**FIGURE 1:**
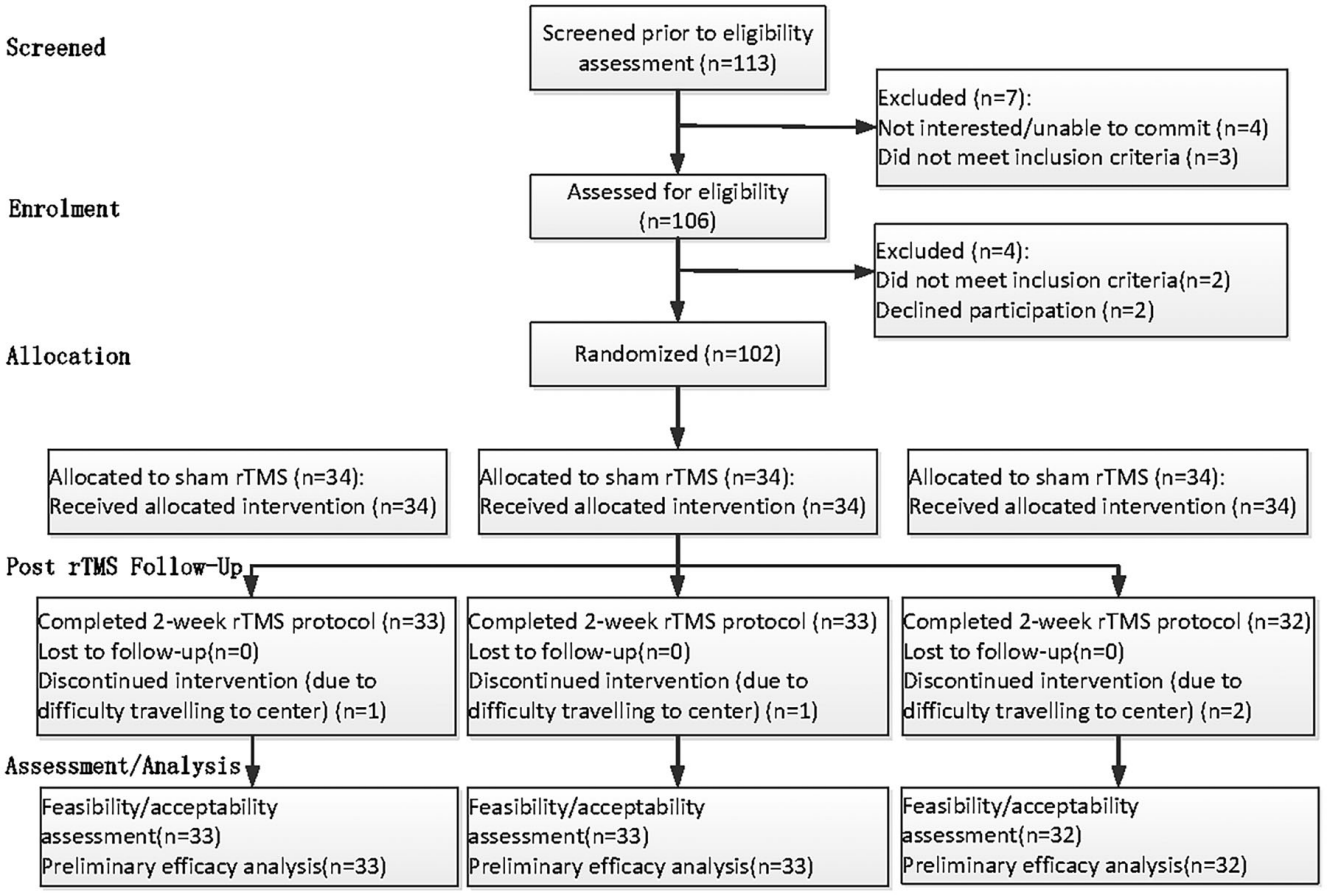
Trial profile. rTMS, repetitive transcranial magnetic stimulation.

This study aimed to determine the efficacy of rTMS at different frequencies in rehabilitating patients with ischemic stroke. The study design and participants are depicted in Figure 1.

### 2.2 Participants

From January 2022 to May 2024, eligible inpatients with ischemic stroke were selected from the Department of Rehabilitation Medicine inpatient wards at the First Affiliated Hospital of South China University. Basic information, including age, gender, disease course, side of the body affected, responsible blood vessel, smoking history, drinking history, hypertension, diabetes, hyperlipidemia, heart disease, atrial fibrillation, location of the lesion, and presence of arterial stenosis, was obtained from hospital visit records. The diagnostic criteria were the points that met the criteria for various cerebrovascular diseases, and computed tomography or magnetic resonance imaging confirmed the presence of a cerebral hemisphere infarction.

The inclusion criteria were as follows: First onset, upper limb Brunnstrom stages II–V, age 50–80 years, disease duration of 2 weeks to 2 months, right‐handedness, stable vital signs, informed consent, and normal cognitive function assessment (Mini‐Mental State Examination score of at least 24) (Jia et al. 2021). First‐onset cases were selected to minimize confounding effects from a previous stroke history, thereby ensuring cohort homogeneity (Chung et al. 2023). This age range (50–80 years) reflects the peak incidence of ischemic stroke (Deng et al. 2012). Right‐handedness dominance is linked to left‐hemisphere motor lateralization, ensuring sample homogeneity (Cazzoli and Chechlacz 2017).

The exclusion criteria were as follows: Implants or lesions contraindicated for rTMS, epilepsy, psychiatric disorders, a history of drugs affecting the central nervous system, and cognitive impairment that prevented cooperation with treatment.

### 2.3 Ethical Consideration

All participants provided informed consent before participating in the trial. This study was approved by the First Affiliated Hospital University of South China Research Ethics Board (Ethics Approval No.: 2021KS‐KF‐13‐01) and registered with Clinicaltrials.gov (ChiCTR2100047637).

### 2.4 Intervention

All patients underwent the same routine rehabilitation treatments, including symptomatic drug treatment and rehabilitation. Rehabilitation treatments included motor relearning therapy, Bobath therapy, Brunnstrom therapy, Rood technique, proprioceptive neuromuscular facilitation technique, occupational therapy, physical factor therapy, acupuncture, and other treatments. The treatment duration was 2 weeks, 6 days per week. The 10 Hz high‐frequency group was given 10 Hz, 100% rMT intensity, and 1800 pulses of rTMS treatment in the M1 area of the central prefrontal cortex on the lesioned side. The 1 Hz low‐frequency group received 1 Hz, 100% rMT intensity, and 1800 pulses of rTMS treatment in the M1 area of the central prefrontal cortex on the lesioned side. High‐frequency rTMS (>5 Hz) is typically used to improve cortical excitability, whereas low‐frequency rTMS (≤1 Hz) is used to inhibit cortical excitability (Valle et al. 2007). The 100% rMT intensity was determined using motor‐evoked potentials (Spampinato et al. 2023). The total pulse count was closely related to the treatment duration and frequency. The sham rTMS group was given sham stimulation intervention, with the coil turned 90° so that its plane was perpendicular to the surface of the skull. All participants were exposed to the same auditory stimuli. All participants received the genuine/sham rTMS intervention once a day, 6 days a week, for 2 weeks. After completion of the trial, the sham rTMS group was compensated with 1 Hz actual rTMS stimulation.

### 2.5 Outcomes

The primary outcome was improved upper limb function after 2 weeks of intervention, as assessed using the Fugl‐Meyer Assessment upper extremity (FMA‐UE) scale. The FMA‐UE assessment is widely used to evaluate sensorimotor upper dysfunction in stroke patients and clinical motor function assessments.

Secondary outcomes and the measurement tools used to assess them included the National Institutes of Health Stroke Scale (NIHSS) and the modified Barthel index scale. The NIHSS is primarily used to evaluate neurological deficits and clinical prognoses of patients. A higher score indicates more severe neurological deficits. Moreover, the modified Barthel index scale is currently the most commonly used scale to assess basic daily living activities, with a higher modified Barthel index score indicating better self‐care ability.

The FMA‐UE includes 33 assessment items on a 3‐point scale. The maximum exercise score is 66 points. The higher the score, the better the upper limb motor function recovery. The severity of the motor impairment can be determined on the basis of the final score. The FMA‐UE assessment score has been repeatedly tested and demonstrated good consistency, responsiveness, and accuracy. The NIHSS is a 15‐item impairment scale used to assess neurological prognosis and recovery in stroke patients. The scale assesses the levels of consciousness, extraocular movements, visual fields, facial muscle function, limb strength, sensory function, coordination, language, speech, and attention. The modified Barthel Index includes 10 items, each with 5 levels and corresponding scores, with a score range of 0–100, including assessment of daily living activities, functional activities, and gait.

### 2.6 Sample Size

The sample size ratio of each group in this study was 1:1:1. The standard deviations of the pre‐ and post‐intervention differences in the FMA‐UE of the sham rTMS, 1 Hz, and 10 Hz groups obtained from the pre‐experiment were 0.52, 0.53, and 0.52, respectively. Assuming an effect size of 0.80 and a significance level of 0.05, the calculation according to the formula demonstrated that 27 subjects were required for each group. Considering a 20% dropout rate during the trial, the sample size required for each group was 27 × (1% + 20%) = 34. After applying the inclusion criteria, each group required a minimum of 34 samples. At the beginning of the trial, the total sample size for the three groups was 113, and 102 patients were selected for the formal trial intervention after screening.

### 2.7 Randomization

Informed consent was obtained by a research doctor. A random number table was created using the Statistical Package for the Social Sciences (SPSS; version 26.0), and the subjects were randomly divided into the sham rTMS group, the 1 Hz on the healthy side group, and the 10 Hz on the affected side group according to their order of appearance in a 1:1:1 ratio. A trained rehabilitation therapist collected basic information and administered the rTMS intervention. The rehabilitation therapist did not participate in any other phase of the trial. A senior rehabilitation therapist conducted the clinical scale assessment and statistical analysis.

### 2.8 Serum Collection

Five milliliters of blood was collected from the patients 12 h after fasting on the day before the intervention and the day after the intervention was completed. The blood was centrifuged at 4°C (4000 rpm for 10 min), and the supernatant was transferred to a 1.5 mL Eppendorf (EP) tube. The samples were then stored at −80°C.

### 2.9 Non‐Targeted Metabolomics Assays

#### 2.9.1 Sample Preparation

Ten samples were randomly selected from each group for metabolomics analysis. The serum samples were removed from the −80°C refrigerator and placed at room temperature to thaw. Approximately 200 µL of the sample was taken, 80 µL of methanol and 400 µL of methyl *tert*‐butyl ether were added, and then extracted using ultrasonication at low temperature for 30 min (5°C, 40 KHz). The sample was then placed in a −20°C refrigerator for 30 min, followed by centrifugation at 13,000 rpm for 15 min at 4°C. The supernatant (350 µL) was transferred to a new EP tube and dried with nitrogen. The supernatant was resuspended in 100 µL of isopropanol:acetonitrile (1:1) extraction solution, vortexed for 30 s, and sonicated at low temperature for 5 min. The mixture was then centrifuged at 13,000 rpm and 4°C for 5 min, and the supernatant was transferred to a sample vial. Approximately 20 µL of the supernatant from each sample was collected, mixed, and vortexed as a QC sample.

#### 2.9.2 Liquid Chromatography–Mass Spectrometry (LC–MS) and Data Analysis

LC–MS analysis was performed using an Agilent 1290 Infinity ultra‐high‐performance LC system coupled with an Agilent 6545 UHD and an Accurate‐Mass Q‐TOF MS. Data were acquired using LC–MS conditions for the samples to be tested. Each sample was analyzed separately in the positive and negative ion modes. Raw data were converted to universal data (mz. data) format using Agilent Masshunter Qualitative Analysis B.08.00 software (Agilent Technologies, USA). The Chromatography Centroid Mass Spectrometry (XCMS) package of R software was used for peak identification, retention time correction, automatic integration, and other pre‐processing tasks. Normalization was performed by dividing the raw peak area by the corresponding internal standard peak area, and the Lipidmaps database (http://www.lipidmaps.org/) was used for qualitative analysis. The edited data matrix was imported into the R software for multivariate statistical analysis.

### 2.10 Statistical Analysis

A chi‐square test was performed on the basic information of the population, such as gender and the side of the body affected by paralysis, using SPSS (version 26.0). All the quantitative data were tested for normality. Measurement results that conform to normal distribution are expressed as mean ± standard deviation (*x* ± *s*). Non‐normal distribution was tested using the non‐parametric rank sum test, and the results are expressed as the median (interquartile range; median [IQR]). A paired *t*‐test was used for intragroup comparisons, and analysis of variance for intergroup comparisons. If the overall difference was statistically significant, the least significant difference test (LSD‐T) method was used for post hoc comparisons to observe differences at each time point. *p* ≤ 0.05 was considered statistically significant. Potential biomarkers were screened using the orthogonal partial least squares discriminant analysis (OPLS‐DA) model's variable importance in the projection (VIP) ≥ 1 and independent sample *t*‐test (*p* < 0.05). A VIP score >1 indicates variables with above‐average importance in the partial least squares discriminant analysis (PLS‐DA) model, ensuring biological relevance, whereas *p* < 0.05 indicates statistical significance, minimizing false positives. The Pearson linear correlation coefficient was used to determine the linear correlation between all differentially expressed metabolites.

## 3 Results

### 3.1 Subject Baseline Characteristics

The intervention was well tolerated by the patients, and no serious adverse events occurred. A total of 113 participants were screened before the eligibility assessment, of which 102 met the inclusion criteria and were enrolled. Four people withdrew from the trial due to an exacerbation of their condition or were transferred to another hospital during the trial, and 98 participants ultimately completed the study. There were no significant differences in baseline characteristics, including age, gender, underlying disease, disease duration (days), and hemiplegic side among the three groups (*p* > 0.05; Table 1). There was a statistically nonsignificant difference between the FMA‐UE, NHISS, and Barthel scores of the three groups before treatment (*p* > 0.05; Table 1).

**TABLE 1:** Baseline clinical findings in participants receiving different repetitive transcranial magnetic stimulations (rTMSs).

Variable	Sham rTMS group (*n* = 33)	1 Hz group (*n* = 33)	10 Hz group (*n* = 33)	*p* value
Age, year mean ± SD	64.3 ± 8.5	59.8 ± 10.0	64.2 ± 10.6	0.104
Gender				0.631
Male (%)	21 (63.6)	24 (72.7)	20 (62.5)	
Female (%)	12 (36.4)	9 (27.3)	12 (37.5)	
Cigarette smoking (%)	11 (33.3)	19 (57.6)	16 (50.0)	0.131
Alcohol (%) Consumption (%)	13 (39.4)	15 (54.5)	16 (50.0)	0.689
Hypertension (%)	27 (81.8)	27 (81.8)	23 (71.9)	0.531
Diabetes mellitus (%)	13 (39.4)	14 (42.4)	13 (40.6)	0.969
Dyslipidemia (%)	9 (27.3)	13 (39.4)	7 (21.9)	0.283
Heart disease (%)	5 (27.3)	3 (3.0)	4 (12.5)	0.018
Disease course, days, median (IQR)	20 (17.0–23.4)	10 (13.6–26.3)	15 (14.7–25.0)	0.302
Right‐sided hemiplegia (%)	15 (45.5)	15 (60.6)	15 (62.5)	0.313
Lesion location				0.973
Cortex (%)	3 (9.1)	3 (9.1)	3 (9.4)	
Subcortical (%)	25 (75.8)	26 (78.8)	26 (81.3)	
Both (%)	5 (15.2)	4 (12.1)	3 (9.4)	
Arterial occlusion (%)	13 (39.4)	20 (60.6)	15 (46.9)	0.217
NIHSS (Mean ± SD)	11.58 ± 7.471	10.33 ± 3.47	9.81 ± 5.227	0.549
FMA‐UE, median (IQR)	11 (4,43.5)	9 (5.5,31.5)	8 (4.3,29)	0.871
Barthel (Mean ± SD)	41.21 ± 24.4	45.15 ± 14.442	46.25 ± 26.640	0.674

Abbreviations: FMA‐UE, Fugl‐Meyer Assessment upper extremity; NIHSS, National Institutes of Health Stroke Scale.

### 3.2 Comparison of Clinical Motor Function Assessments

#### 3.2.1 FMA‐UE Score Before and After rTMS Treatment at Different Frequencies

After treatment, FMA‐UE scores of the three groups were significantly higher than those before the intervention in each group, and the difference was statistically significant (*p* < 0.05; Table 2).

**TABLE 2:** Fugl‐Meyer Assessment upper extremity (FMA‐UE) score before and after treatment (points), median (interquartile range [IQR]).

	Sham rTMS group (*n* = 33)	1 Hz group (*n* = 33)	10 Hz group (*n* = 32)
Baseline	11 (4,43.5)	9 (5.5,31.5)	8 (4.3,29)
2 weeks later	14 (7,45.5)	22 (12.5,45.5)	14.5 (8.3)
*Z*‐score	−4.743	−5.016	−4.866
*p* value	0.000	0.000	0.000

Abbreviation: rTMS, repetitive transcranial magnetic stimulation.

#### 3.2.2 NIHSS Score Before and After rTMS Treatment at Different Frequencies

After treatment, the NIHSS scores of the three groups were significantly lower than those before treatment in each group, and the difference was statistically significant (*p* < 0.05; Table 3).

**TABLE 3:** National Institutes of Health Stroke Scale (NIHSS) score before and after treatment (points), mean ± SD.

	Sham rTMS group (*n* = 33)	1 Hz group (*n* = 33)	10 Hz group (*n* = 32)
Baseline	11.58 ± 7.471	10.33 ± 3.47	9.81 ± 5.227
2 weeks later	10.06 ± 7.167	6.09 ± 2.674	6.97 ± 4.638
*T*‐score	8.671	9.423	9.131
*p* value	0.000	0.000	0.000

Abbreviation: rTMS, repetitive transcranial magnetic stimulation.

#### 3.2.3 Barthel Score Before and After rTMS Treatment at Different Frequencies

After treatment, Barthel index scores of the three groups were significantly higher than before treatment, and the difference was statistically significant (*p* < 0.05; Table 4).

**TABLE 4:** Modified Barthel score before and after treatment (points), mean ± SD.

	sham rTMS group (*n* = 33)	1 Hz group (*n* = 33)	10 Hz group (*n* = 32)
Baseline	41.21 ± 24.4	45.15 ± 14.442	46.25 ± 26.640
2 weeks later	48.79 ± 25.001	65.91 ± 16.931	59.84 ± 24.544
*T*‐score	−12.217	−14.527	−9.792
*p* value	0.000	0.000	0.000

Abbreviation: rTMS, repetitive transcranial magnetic stimulation.

#### 3.2.4 Comparison of the Difference in Scores on Each Scale Before and After rTMS Treatment at Different Frequencies

The pre‐ and post‐intervention FMA‐UE scale scores exhibited statistically significant differences between the three groups (*p* < 0.05). The pre‐ and post‐intervention Barthel scale scores exhibited statistically significant differences between the three groups (*p* < 0.05). For the NIHSS score, the two groups that received rTMS intervention exhibited statistically significant differences before and after the intervention (*p* < 0.05) compared with the sham rTMS group, whereas the difference in the 1 and 10 Hz groups before and after the intervention was statistically nonsignificant (*p* > 0.05; Table 5). These findings demonstrate that 1 and 10 Hz rTMS were more effective than sham rTMS in improving upper limb function and activities of daily living (ADL) in patients with ischemic stroke. Notably, the therapeutic benefits of 1 Hz rTMS on upper limb motor recovery and ADL appear to be superior to those of 10 Hz rTMS.

**TABLE 5:** Comparison of the difference in scale scores before and after treatment (points), median (interquartile range [IQR]).

	Sham rTMS group (*n* = 33)	1 Hz group (*n* = 33)	10 Hz group (*n* = 32)	Sham rTMS group vs. 1 Hz group *p* value	sham rTMS group vs. 10 Hz group *p* value	1 Hz group vs. 10 Hz group *p* value
FMA‐UE	3 (2,4)	8 (5,15.5)	5 (2.25,8)	0.000	0.006	0.014
NIHSS	1 (1,2)	5 (2.5,7)	3 (2,4)	0.000	0.009	0.159
Barthel	5 (5,10)	20 (15,25)	12.5 (5,20)	0.000	0.007	0.005

Abbreviations: FMA‐UE, Fugl‐Meyer Assessment upper extremity; NIHSS, National Institutes of Health Stroke Scale; rTMS, repetitive transcranial magnetic stimulation.

### 3.3 Non‐Targeted Lipidomics

**FIGURE 2:**
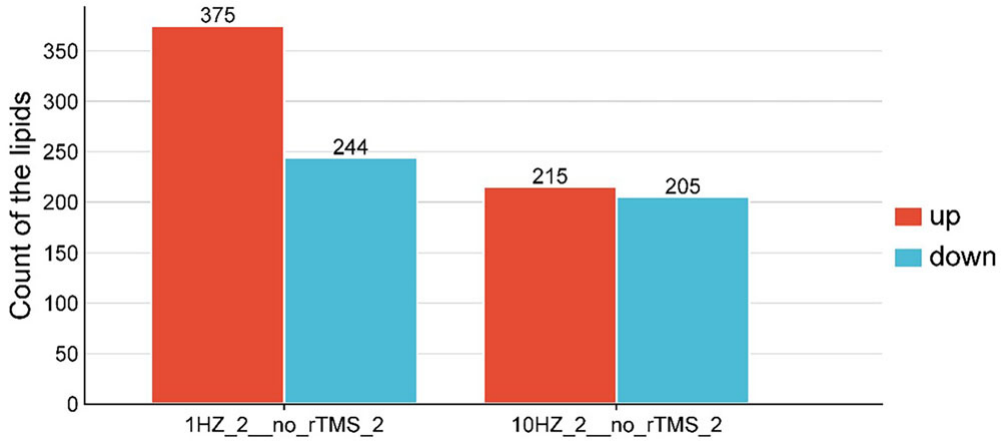
Total number of lipid differential metabolites identified. 1HZ_2 represents the 1 Hz group after treatment; no_rTMS_2 represents the sham rTMS group after 2 weeks of intervention; 1HZ_2 represents the 1 Hz rTMS group after 2 weeks of intervention; 10HZ_2 represents the 10 Hz rTMS group after 2 weeks of intervention.

A serum lipidomic analysis was performed to investigate the effects of rTMS intervention on blood lipid levels in patients with ischemic stroke. The VIP value and independent sample *t*‐test were used to identify differentially expressed metabolites. A metabolite was considered statistically significant when VIP > 1 and *p* < 0.05. Among them, 619 significantly different metabolites were identified in the 1 Hz group compared with the sham rTMS group (1HZ_2__no_rTMS_2); a total of 375 were upregulated, and 244 were downregulated (Figure 2). The 10 Hz group was compared with the sham rTMS group (10HZ_2__no_rTMS_2), and 420 significantly different metabolites were identified, with 215 upregulated and 205 downregulated.

**FIGURE 3:**
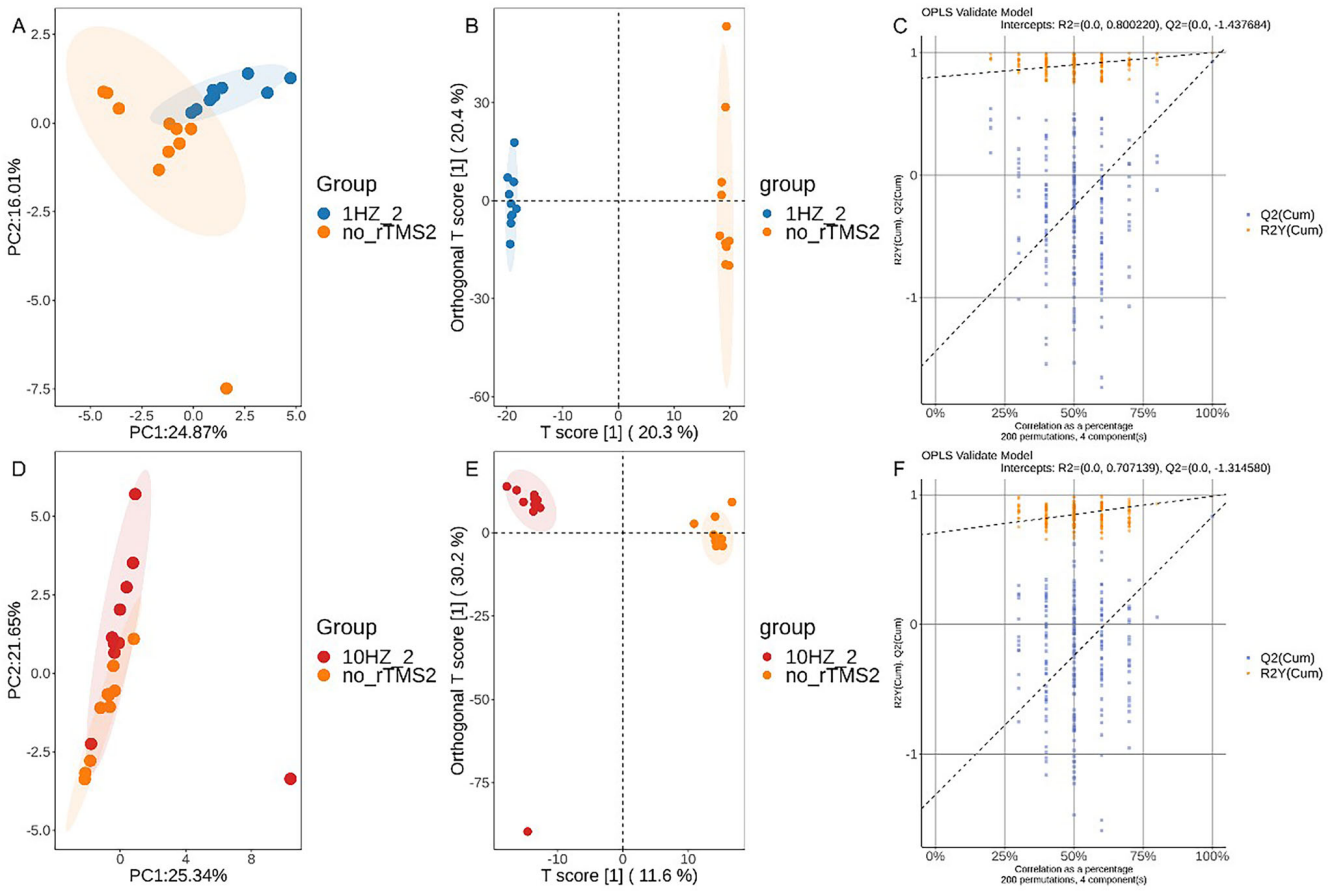
Multivariate statistical analysis of the 1 and 10 Hz groups compared with the sham rTMS groups after treatment. (A and D) Scatter plots of lipid spectrometry PCA scores in the 1 and 10 Hz groups after treatment compared with the sham rTMS group; (B and E) scatter plots of PLS‐DA scores of lipid spectra in the 1 and 10 Hz groups after treatment compared with the sham rTMS group; (C and F) lipid spectrometry RPT test arrangement charts of the 1 and 10 Hz groups compared with the sham rTMS group after treatment. 1HZ_2 represents the 1 Hz group after treatment; no_rTMS_2 represents the sham rTMS group after treatment; 10HZ_2 represents the 10 Hz group after treatment. OPLS, orthogonal partial least squares.

We then performed principal component analysis (PCA) and PLS‐DA. PCA is an unsupervised modeling method that provides an overview of the grouping patterns. Simultaneously, PLS‐DA, a supervised statistical model for multivariate analysis, helps identify the most influential distinguishing features. The PCA results revealed that the separation effect in the sham rTMS and 1 Hz groups was better than that in the sham rTMS and 10 Hz groups, indicating that low‐frequency rTMS intervention has a specific regulatory effect on lipid metabolism after stroke (Figure 3A,D). However, PLS‐DA results revealed significant intergroup differences between the sham rTMS and 1 Hz groups, as well as between the sham rTMS and 10 Hz groups after treatment (Figure 3B,E). In our model, the validation results after treatment of the sham rTMS and 1 Hz groups demonstrated *R* ^2^ = 0.80, *Q* ^2^ = −1.437, indicating that this model exhibits strong interpretability and can explain the intergroup differences between the two groups better. The validation results after treatment of the sham rTMS and 10 Hz groups revealed *R* ^2^ = 0.707, *Q* ^2^ = −1.314, indicating that this model is highly interpretable and can better explain the group differences between the two groups (Figure 3C,F). The regression lines of *Q* ^2^ exhibited a negative intercept, *Q* ^2^ < 0, indicating that this model was not overfitted.

**FIGURE 4:**
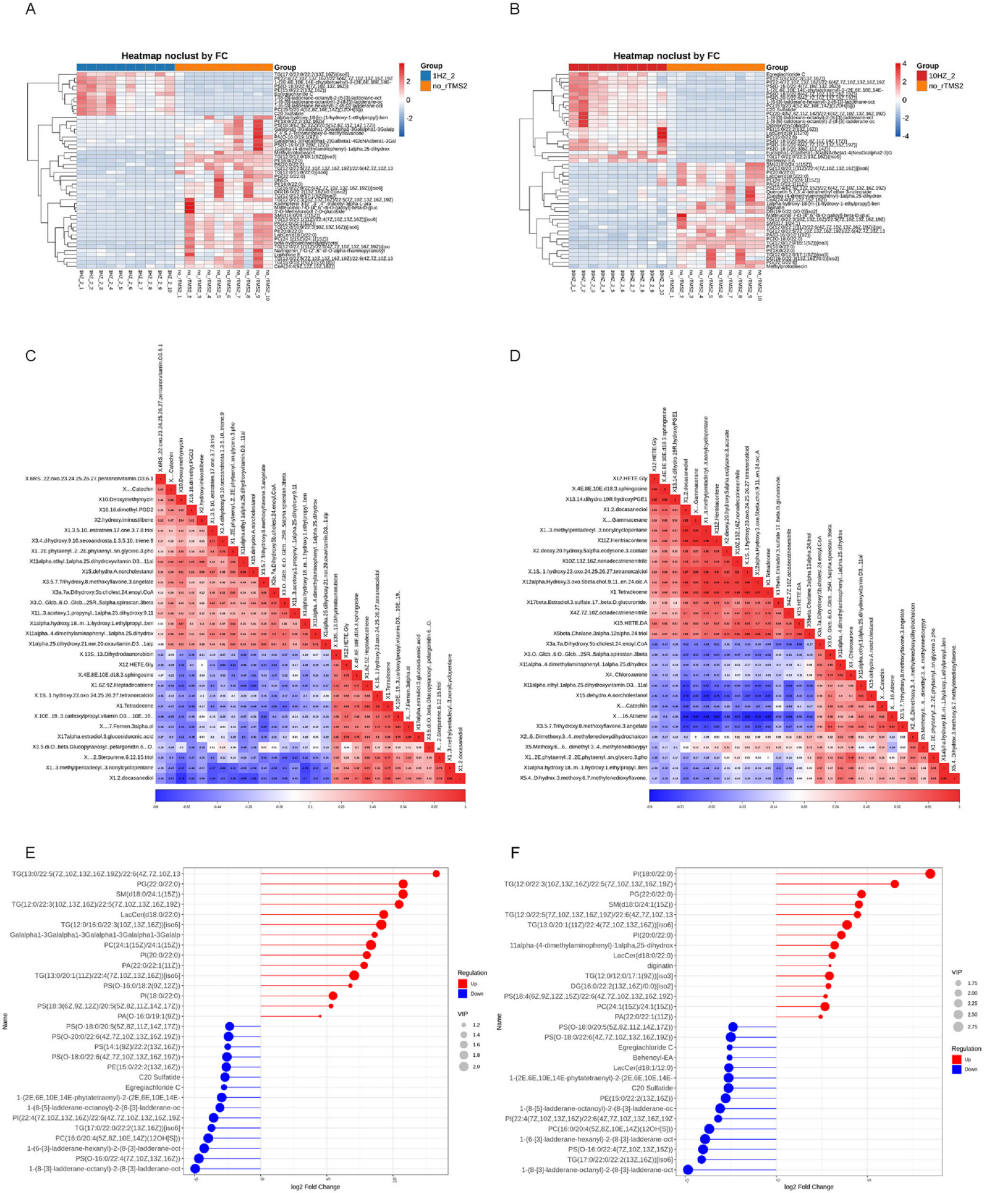
Differential metabolite heatmaps of the 1 and 10 Hz groups compared with the sham rTMS groups after treatment. (A and B) Differential lipid metabolite profiles in the 1 and 10 Hz groups after treatment compared with those in the sham rTMS group; (C and D) differential lipid metabolite linear correlation heatmap of the 1 and 10 Hz groups compared with the sham rTMS group after treatment; (E and F) matchgrams of lipid spectra in the 1 and 10 Hz groups compared with the sham rTMS group after treatment. No _rTMS_2 represents the sham rTMS group after treatment, 1HZ_2 represents the 1 Hz group after treatment, and 10HZ_2 represents the 10 Hz group after treatment.

Subsequently, lipids with significant differences between the different groups were visualized using a heatmap. After treatment, the sham rTMS group exhibited higher expression abundances of triglyceride and sphingolipid metabolism and lower expression abundances of acylglycerol metabolism (Figure 4A). The 1 Hz group exhibited higher acylglycerol metabolism and lower triglyceride and sphingolipid metabolism after treatment (Figure 4B).

The linear correlation analysis of the differential metabolites revealed that clavulanic acid, pentanedione, vitamin D3, and flavonoids were positively correlated. The negatively correlated metabolites included vitamin D3, triol, 1‐tetradecene, 1,2‐docosanol, and heptadecatriene. The metabolites with a positive correlation were flavonoids and choline phosphate, whereas the metabolites exhibiting a negative correlation were vitamin D3, catechin, 16‐terpenes, and 3,5,7‐trihydroxy‐8‐methoxyflavone 3‐angelate (Figure 4C,D).

Metabolite screening: The heatmap demonstrated that compared with the sham rTMS group, the 1 Hz group exhibited upregulated diacylglycerol phosphorylcholine, triacylglycerol, ceramide phosphorylcholine, diacylglycerol phosphorylcholine, diacylglycerol phosphorylglycerol, diacylglycerol phosphate, and 2‐acylglycerol phosphate, and downregulated diacylglycerol (DAG; Figure 4E). Compared with the sham rTMS group, diacylglycerol phosphorylcholine and triacylglycerol were significantly upregulated, and DAG was significantly downregulated in the 10 Hz group (Figure 4F).

**FIGURE 5:**
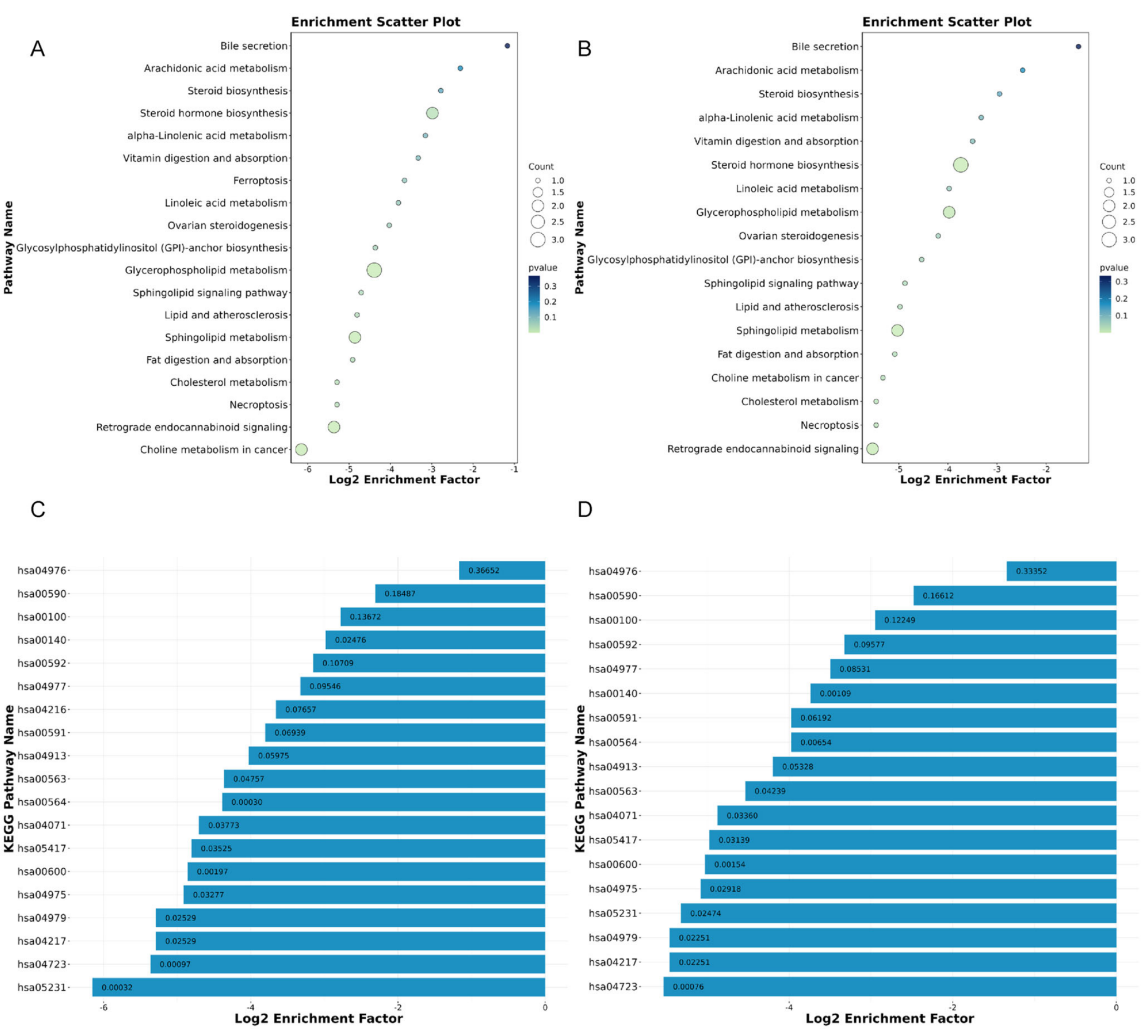
Advanced analysis of differential metabolites in the 1 and 10 Hz groups after treatment compared with the sham rTMS group. (A and C) The top 20 signal pathways and *p* values in the 1 Hz group after treatment compared with the sham rTMS group; (B and D) the top 20 signal pathways and *p* values in the 10 Hz group after treatment compared with the sham rTMS group. No_rTMS_2 represents the sham rTMS group after treatment, 1HZ_2 represents the 1 Hz group after treatment, and 10HZ_2 represents the 10 Hz group after treatment.

Compared with sham rTMS, the signal pathways with significant enrichment of differential metabolites after 1 Hz low‐frequency rTMS intervention included intra‐tumoral choline metabolism, the retrograde endocannabinoid system, necroptosis, cholesterol metabolism, fat digestion and absorption, sphingolipid metabolism, lipids and atherosclerosis, glycerophospholipid metabolism, and glycosylphosphatidylinositol anchor biosynthesis (Figure 5A,B). Compared with the sham rTMS group, the signal pathways with significant enrichment of differential metabolites after 10 Hz high‐frequency rTMS intervention included the retrograde endocannabinoid system, necroptosis, cholesterol metabolism, intra‐tumoral choline metabolism, fat digestion and absorption, sphingolipid metabolism, lipids and atherosclerosis, sphingolipids, and glycerophospholipid‐anchoring protein biosynthesis (Figure 5C,D).

## 4 Discussion

Few studies have investigated the relationship between different TMS frequencies and changes in serum lipid metabolism after ischemic stroke. This study aimed to evaluate the effects of commonly used rTMS protocols on upper limb motor function and lipid metabolism in patients with ischemic stroke and to investigate the potential targets and mechanisms of rTMS in the treatment of ischemic stroke. Our results demonstrated that 1 Hz rTMS on the affected side and 10 Hz rTMS on the unaffected side significantly improved upper limb motor function, neurological function, and ADL in ischemic stroke patients and that 1 Hz rTMS was more effective than 10 Hz rTMS in improving upper limb motor function. Moreover, a reliable untargeted lipidomics method was used to exclude the influence of internal and external factors, establish a stable model, screen for differential metabolites related to rTMS recovery in patients with ischemic stroke, and enrich them with the corresponding metabolic pathways. The results demonstrated that after rTMS treatment at different frequencies, diacylglycerol phosphatidylinositol and triacylglycerol were upregulated, whereas DAG was downregulated. These three metabolites may be potential targets for rTMS to promote recovery after ischemic stroke. Additionally, the retrograde endocannabinoid system, choline metabolism, necroptosis, cholesterol metabolism, atherosclerosis, and glycerophospholipid metabolic pathways were identified to play essential roles in lipid metabolism during rTMS treatment of ischemic stroke.

Limb movement dysfunction is the most common sequela of ischemic stroke (Zhao et al. 2022). Impaired consciousness, vision, speech, and daily living skills are also difficulties that patients with ischemic stroke face and need to overcome. rTMS, a new, noninvasive, and long‐lasting method, has been widely used in stroke rehabilitation. However, the choice between low frequency on the healthy side and high frequency on the affected side remains controversial (Bai et al. 2022; Liu et al. 2021; Xia et al. 2022). Moreover, the results revealed that after 2 weeks of intervention, sham rTMS, 1 Hz rTMS, and 10 Hz rTMS could improve patients’ neurological function, upper limb function, and self‐care abilities. This indicates that in the early stages of ischemic stroke, routine manual therapy and rTMS intervention can play a protective role in patients’ neurological function, upper limb function, and daily living ability (Guan et al. 2017). Besides, 1 and 10 Hz rTMS significantly improved the FMA‐UE, NHISS, and Barthel scores before and after ischemic stroke treatment, which was better than sham rTMS. Both low‐ and high‐frequency rTMS can improve motor function by modulating motor cortex activation in the early stages of stroke (Du et al. 2019), consistent with our results. The 1 Hz rTMS protocol was significantly superior to the 10 Hz rTMS protocol regarding changes in FMA‐UE and Barthel scores before and after ischemic stroke treatment. A meta‐analysis revealed that the current evidence supports the role of inhibitory stimulation in improving the excitability of the affected M1 cortex (Bai et al. 2022). However, the difference in NHISS scores before and after treatment with 1 and 10 Hz TMS was statistically nonsignificant, consistent with Chen's study (Chen, Shen et al. 2021).

Lipidomics is the comprehensive analysis of lipids in organisms using high‐throughput analysis techniques combined with bioinformatics tools (Züllig et al. 2020). Our differential metabolite analysis revealed that after rTMS treatment, the serum triacylglycerol levels of patients with ischemic stroke were upregulated, whereas DAG was downregulated. DAG is a secondary messenger in the cell membrane. When specific receptors on the cell membrane are stimulated to change their conformation, phosphoinositide hydrolysis is induced, generating DAG and triphosphoinositide. Inositol phosphate metabolism may affect neuronal death (Berridge 2016). An exogenous supply of a small amount of phosphatidylinositol 4,5‐bisphosphate can rescue the functional congestion of the damaged brain in a mouse model of cerebral autosomal dominant arteriopathy with subcortical infarction and leukoencephalopathy (Dabertrand et al. 2021). We discovered that both low‐ and high‐frequency rTMS interventions reversed the downregulation of phosphatidyl inositol after ischemic stroke. DAG can be converted to triacylglycerol through an acylation reaction (Chen, Harwood et al. 2022), a risk factor for ischemic stroke (Shi et al. 2021).

The lipid metabolism analysis revealed that the differential metabolites after rTMS intervention were enriched in the retrograde endocannabinoid system, choline metabolism, necroptosis, cholesterol metabolism and atherosclerosis, glycerophospholipid metabolism, and sphingolipid metabolism. The endocannabinoid system is essential to the nervous system (Cristino et al. 2020; Estrada and Contreras 2020). Studies have revealed that the endocannabinoid system reduces inflammation and damage to brain tissue after stroke (Vicente‐Acosta et al. 2022) and promotes remyelination to exert a neuroprotective effect (Atalay et al. 2019; Tomas‐Roig et al. 2020). Clinical studies have demonstrated that improved choline metabolism can promote neurological function repair after stroke (Sagaro and Amenta 2023). Activation of necroptosis releases inflammatory mediators, further aggravating brain damage (Nikseresht et al. 2019; Vicente‐Acosta et al. 2022). Necroptosis inhibitors exhibit inhibitory effects on cerebral ischemic injury and neuroinflammation (Deng et al. 2019). rTMS‐targeted necroptosis treatment strategies are considered a potential new approach for stroke treatment. Atherosclerosis is the most common risk factor for ischemic stroke (Jacob et al. 2022). Plaque instability may induce thrombosis, leading to an acute ischemic stroke (Ng 2022). High cholesterol levels not only contribute to atherosclerosis, which indirectly increases the risk of stroke, but may also directly increase the risk of stroke by affecting the stability and function of the brain blood vessels (Gaba et al. 2023; Prasad and Mishra 2022). Downregulating cholesterol levels has been demonstrated to effectively reduce the risk of stroke, especially in patients with evidence of atherosclerosis (Lee et al. 2022). Inhibiting the expression of atherosclerosis and cholesterol may be a potential research direction for the rTMS treatment of ischemic stroke. The components of glycerophospholipids occupy a vital position in the brain tissue (Sarkar and Lipinski 2024). Studies have demonstrated that increased expression of glycerophospholipids can improve endothelial function and reduce atherosclerosis in the carotid arteries of middle‐aged and older people, thereby reducing the incidence of stroke (DeVan et al. 2016).

Overall, low‐ and high‐frequency rTMS significantly improved upper limb motor function in patients with ischemic stroke, with the effect of low‐frequency rTMS being better than that of high‐frequency rTMS. The mechanism by which rTMS improves ULMD may be related to the regulation of the metabolism of lipid molecules, including diacyl glycerophosphocholine, triacylglycerol, and diglyceride.

Furthermore, the lipid metabolism pathways were summarized using the Kyoto Encyclopedia of Genes and Genomes analysis. Although we identified specific plasma lipids/lipid pathways associated with rTMS treatment for ischemic stroke, this study was mainly focused on correlation analysis and could not determine the causal relationships. Our study had a relatively small sample size, and the intervention period was only 2 weeks. However, these findings are expected to guide future animal and clinical studies to determine the mechanism of metabolic responses to rTMS treatment for ischemic stroke. The consideration of different rTMS treatment protocols will add value to efforts for upper limb functional rehabilitation interventions in patients with stroke. Our results are expected to support the use of novel bioanalytical tools, including MS‐based lipidomics, to uncover the complex lipid regulatory processes involved in rTMS treatment of ischemic stroke patients.

## Author Contributions

**Meng‐Meng Li**: conceptualization, methodology, writing–original draft. **Fei‐Yang Jia**: data curation, formal analysis, writing–original draft. **Peng‐Cheng Liu**: investigation, resources. **Hong‐Ya Liu**: supervision, funding acquisition. **Gui‐Juan Zhou**: software, funding acquisition. **Xin‐Ke Peng**: validation, funding acquisition. **Jin‐Ling Wang**: investigation, project administration. **Shu‐Zhi Li**: formal analysis, resources. **Jing Liu**: supervision, writing–review and editing. **Jun Zhou**: funding acquisition, supervision, writing–review and editing.

## Conflicts of Interest

The authors declare no conflicts of interest.

### Peer Review

The peer review history for this article is available at https://publons.com/publon/10.1002/brb3.70558

## Data Availability

The data that support the findings of this study are available from the corresponding author upon reasonable request.
